# Comparative analysis of the *Burkholderia cenocepacia* K56-2 essential genome reveals cell envelope functions that are uniquely required for survival in species of the genus *Burkholderia*

**DOI:** 10.1099/mgen.0.000140

**Published:** 2017-11-21

**Authors:** April S. Gislason, Keith Turner, Mike Domaratzki, Silvia T. Cardona

**Affiliations:** ^1^​ Department of Microbiology, University of Manitoba, Winnipeg, MB, R3T 2N2, Canada; ^2^​ Monsanto Company, 700 Chesterfield Parkway W, Chesterfield, MO, 63017, USA; ^3^​ Department of Computer Science, University of Manitoba, Winnipeg, R3T 2N2, Canada; ^4^​ Department of Medical Microbiology & Infectious Diseases, University of Manitoba, Winnipeg, MB, R3E 0J9, Canada

**Keywords:** *Burkholderia*, essential genes, transposon mutagenesis, Tn-seq, antibiotic targets, cell envelope

## Abstract

*Burkholderia cenocepacia* K56-2 belongs to the *Burkholderia cepacia* complex, a group of Gram-negative opportunistic pathogens that have large and dynamic genomes. In this work, we identified the essential genome of *B. cenocepacia* K56-2 using high-density transposon mutagenesis and insertion site sequencing (Tn-seq circle). We constructed a library of one million transposon mutants and identified the transposon insertions at an average of one insertion per 27 bp. The probability of gene essentiality was determined by comparing of the insertion density per gene with the variance of neutral datasets generated by Monte Carlo simulations. Five hundred and eight genes were not significantly disrupted, suggesting that these genes are essential for survival in rich, undefined medium. Comparison of the *B. cenocepaci*a K56-2 essential genome with that of the closely related *B. cenocepacia* J2315 revealed partial overlapping, suggesting that some essential genes are strain-specific. Furthermore, 158 essential genes were conserved in *B. cenocepacia* and two species belonging to the *Burkholderia pseudomallei* complex, *B. pseudomallei* K96243 and *Burkholderia thailandensis* E264. Porins, including OpcC, a lysophospholipid transporter, LplT, and a protein involved in the modification of lipid A with aminoarabinose were found to be essential in *Burkholderia* genomes but not in other bacterial essential genomes identified so far. Our results highlight the existence of cell envelope processes that are uniquely essential in species of the genus *Burkholderia* for which the essential genomes have been identified by Tn-seq.

## Abbreviations

Bcc, Burkholderia cepacia complex; CF, cystic fibrosis; COG, cluster of orthologous group; DEG, database of essential genes; HDTM, high-density transposon mutagenesis; ORF, open reading frame; LPS, lipopolysaccharide; PrhaB, rhamnose-inducible promoter; Tn-seq, transposon sequencing; UID, unique insertion density.

## Data Summary

1. The Illumina sequencing reads generated and analysed during the current study are available in the NCBI Sequence Read Archive (SRA) repository, accession number SRP112587, https://www.ncbi.nlm.nih.gov/sra/?term=SRP112587.

2. The script used to identify and trim reads containing the transposon sequence and map the genomic regions can be accessed at https://github.com/khturner/Tn-seq/blob/master/TnSeq2.sh.

3. Custom scripts used to determine gene essentiality can be accessed at https://github.com/khturner/Tn-seq/tree/dockerize.

## Impact Statement

The large and dynamic genomes of *Burkholderia* are likely linked to their profound adaptability and pathogenicity. The differences found in the genomes of closely related *Burkholderia* strains suggest that their essential genomes may also vary. In this work, we used high-density transposon insertion sequencing to predict that the essential genome of *Burkholderia cenocepacia* K56-2 consists of more than five hundred genes. Despite *B. cenocepacia* K56-2 and J2315 belonging to the same clonal complex, comparison of their essential genomes revealed partial divergence. Further comparative analysis with the essential gene sets of two *Burkholderia pseudomallei* group pathogens identified 158 essential genes that are conserved among the four *Burkholderia* strains. Three genes within this common essential gene set encode proteins that contribute to the integrity of the cell envelope and were identified as uniquely essential in the three species of the genus *Burkholderia*. We propose that the normally non-essential functions of intrinsic antimicrobial resistance and cell envelope impermeability that characterize the genus *Burkholderia* are mechanisms tied to the survival of the species of the genus *Burkholderia* that were analysed.

## Introduction

Determining the essential genomes of bacteria has furthered our understanding about the fundamental processes required for survival [1–6] and provided a first step in identifying putative targets for developing antibacterial therapies [7–9]. The identification of essential genes is challenging due to the lethal phenotype that mutagenesis of essential genes cause. Yet, identification of essential genes in laboratory conditions can be achieved by recovering mutants growing in rich, undefined medium after concerted disruption of non-essential genes [10–13]. Next generation sequencing has facilitated the use of saturated transposon mutagenesis to identify the essential genomes of many bacteria. To identify essential genes from a high-density transposon mutant (HDTM) library, the genomic DNA from the library is isolated and the transposon–genome junctions are selectively amplified by PCR and then sequenced. Transposon insertion sites are then identified by mapping the reads to the genome. Identification of transposon insertion sites in HDTM libraries has resulted in the successful identification of the essential genomes in many different bacteria [14–18]. Different methods for creating, sequencing and analysing HDTM libraries have been developed including HITS [19], INSeq [20], TraDIS [15], transposon sequencing (Tn-seq), and Tn-seq circle [21]. The Tn-seq circle method was developed to improve the recovery of transposon-containing reads by selective circularization of single-stranded DNA containing the transposon sequence and subsequent digestion of extraneous genomic DNA [21]. Different statistical frameworks can be applied for essential gene identification [14, 22, 23]. By considering the abundance of each clone in the library, the degree of the essentiality of a gene can be determined. A commonly used analysis to determine whether a gene is essential is by allocating a value per gene that is representative of insertion site density or depth of reads mapping to the gene, divided by the gene’s length [15]. The resulting distribution of values for all genes is bimodal. Essential and non-essential genes can then be distinguished by a point that separates the two distributions. While this approach has been successful in predicting the essential genomes of bacteria [15, 23], it does not account for the random variance of insertion density in the genome and requires a highly saturated transposon mutant library to identify essential genes. A recently developed data analysis pipeline compares the abundance of each transposon mutant in the library with the variance of a neutral dataset generated by Monte Carlo simulations to determine the probability that a gene is essential [16], increasing the confidence of the essential gene prediction.

The *Burkholderia pseudomallei* and *Burkholderia cepacia* complex (Bcc) groups contain human pathogens characterized by their intrinsic antibiotic resistance [24]. Members of the *B. pseudomallei* group include *B. pseudomallei*, *Burkholderia mallei*, *Burkholderia thailandensis, Burkholderia oklahomensis and Burkholderia humptydooensis* [25–27]. *B. pseudomallei* is the most virulent species of the genus *Burkholderia* and is the causative agent of melioidosis [28]. The Bcc includes at least 20 phenotypically similar, but genetically distinct species [29–33]. Several members of the Bcc cause severe lung infections in cystic fibrosis (CF) patients [34]. *Burkholderia cenocepacia* K56-2 is a Bcc strain of the epidemic clonal complex 31 [35, 36], which was isolated from a CF patient in Canada. *B. cenocepacia* K56-2 is from the same clonal complex as the European strain J2315, but is more virulent than J2315 in zebra fish and *Caenorhabditis elegans* host models [37, 38] and has been used extensively in the development of molecular genetic tools for Bcc [39]. While the genome of *B. cenocepacia* J2315 has been completely sequenced, revealing three chromosomes and a plasmid, [40] there is only a draft genome available for *B. cenocepacia* K56-2 [41]. The adaptability of *B. cenocepacia* to the CF lung has been attributed to the plasticity of the genome, which is evident by the presence of genomic islands and insertion sequences [40, 42–44]. All but a few species of the genus *Burkholderia* are particularly susceptible to integration of foreign DNA as they do not possess CRISPR-Cas systems [45–47]. This trend increases the likelihood for gene redundancy and the differential assignment of a gene as essential or as part of the accessory genome for species of the genus *Burkholderia*.

In this work, we used the Tn-seq circle method [21] and the data analysis pipeline using a Monte Carlo simulation-based method [16] to identify the essential genome of *B. cenocepacia* K56-2. To date, the essential genomes of three species of *Burkholderia, B. pseudomallei* [22], *B. thailandensis* [48] and *B. cenocepacia* [23] have been identified, but no comparative analysis has been performed on the common essential functions. We identified 158 essential genes that are common in four *Burkholderia* essential genomes. Moreover, porins, a lysophospholipid transporter, and a protein involved in polymyxin resistance are essential in the three species of *Burkholderia* analysed but not in any other bacterial essential genome described so far. Our work underscores particular aspects of the cell envelope that confer antibiotic resistance as uniquely essential functions in three species of the genus *Burkholderia*.

## Methods

### Bacterial strains and growth conditions

The bacterial strains and plasmids used in this study are listed in Table S1 (available in the online Supplementary Material)*. B. cenocepacia* K56-2, a closely related strain of *B. cenocepacia* J2315 and isolated from a CF patient [49], was the strain used in this study. All bacteria were grown in Luria-Bertani (LB) media (BD) at 37 °C with shaking at 220 r.p.m. in a New Brunswick Scientific E24 shaking incubator for broth cultures unless otherwise indicated. *Escherichia coli* strains were grown in 40 µg ml^−1^ kanamycin (Kan40) or 50 µg ml^−1^ trimethoprim (Tp50) when appropriate. *B. cenocepacia* transposon mutants were selected for in 100 µg ml^−1^ trimethoprim (Tp100), 50 µg ml^−1^ gentamicin (Gm50), and 0.2 % rhamnose (0.2 % rha). Additionally, the transposon mutants were grown in Tp100 with or without 0.2 % rha when appropriate. Growth was estimated by measuring the OD_600_ using a Biotek Synergy 2 plate reader. All chemicals were ordered from Sigma Chemical Co. unless otherwise indicated.

### Production of the one million high-density transposon mutant (HDTM) library

Triparental matings were carried out to conjugate pRBrhaBoutgfp into wild-type *B. cenocepacia* K56-2. This was done using an *E. coli* SY327 donor strain [50] and an *E. coli* MM290 helper strain carrying the pRK2013 plasmid [51]. *E. coli* SY327 and MM290 were plated on LB agar supplemented with Tp50 or Kan40, respectively. *B. cenocepacia* K56-2 was grown in LB broth. All three strains were grown for 16–18 h at 37 °C. Afterwards, *B. cenocepacia* K56-2 was subcultured in 5 ml LB broth at 37 °C until an OD_600_ of 0.3–0.6 was reached. Colonies of *E. coli* SY327 and MM290 were collected and resuspended in 5 ml LB in separate snap-cap tubes. From each of the *E. coli* SY327 and MM290 suspensions, volumes equivalent to 1.5 ml with an OD_600_ of 0.3 were mixed with *B. cenocepacia* suspension equivalent to 0.5 ml with an OD_600_ of 1.0. The mixture was vortexed then centrifuged for 1 min at 6000 r.p.m. The pellet was resuspended, then approximately seventy 20–25-µl aliquots of the triparental mating were spotted on LB agar plates containing 0.2 % rha, allowed to dry and incubated at 37 °C for 2 h. Bacteria from the triparental mating spots were combined and resuspended in 6–7 ml LB broth. Aliquots of 500 µl of bacteria were plated onto 8–10 Q-trays containing LB agar with 0.2 % rha, Tp100 and Gm50, and incubated for 48 h. After incubation, pools of approximately 20 000 bacterial cells were collected into 5 ml LB with 20 % glycerol (v/v) and stored at −80 °C in 400 µl aliquots. Nine triparental mating experiments were performed to produce an HDTM library of approximately 1 000 000 mutants. To create the sequencing-ready HDTM library, glycerol stocks from each triparental mating were pooled together such that there was equal representation of each transposon mutant.

### Molecular biology techniques

The DNA concentration of the samples was measured using the Qubit dsDNA BR Assay kit (Invitrogen). DNA (1–2 µl) was added to Qubit dsDNA BR working solution such that the total volume was 200 µl. The mixture was vortexed for 2–3 s, ensuring that no bubbles formed, and was then incubated for 2–5 min at room temperature prior to the measurement of DNA concentration with a Qubit 2.0 fluorometer (Invitrogen).

DNA purification of amplicons was carried out using the Agencourt AMPure XP system (Beckman Coulter). Room temperature AMPure XP beads were mixed with DNA samples in low DNA-binding microcentrifuge tubes (Eppendorf), after which the mixtures were incubated for 5 min at room temperature. The tubes were then placed next to the magnets on the magnet rack to sit until all the beads were pulled towards the magnet. The supernatant was then removed, after which the beads were washed twice with 1 ml of 80 % ethanol while the tubes were still on the rack. After the supernatant was removed, the beads were allowed to air dry for 5 min on the magnet to allow evaporation of excess ethanol. The tubes were removed from the magnets, and DNA was eluted with EB buffer (Qiagen) The DNA samples were quantified after purification.

### Genomic DNA isolation

Cell suspensions (0.5–1 ml) were washed twice, first in 500 µl 0.85 % saline followed by 500 µl TES (10 mM Tris, 30 mM EDTA, 150 mM NaCl in milliQ H_2_O). Afterwards, the pellet was resuspended in 250 µl T10E25 (25 mM Tris, 62.5 mM EDTA in milliQ H_2_O). Fifty microlitres of 2 mg ml^−1^ lysozyme solution and 1 µl of 100 mg ml^−1^ RNase (Qiagen) were added and then incubated for 15 min at 37 °C. Next, 60 µl sarcosyl-protease solution (19.5 mM Tris, 0.975 mM EDTA, 100 mg ml^−1^ sarcosyl, 5 g ml^−1^ protease K in milliQ H_2_O) was added followed by incubation for 16–18 h at 37 °C. After incubation, the suspension was mixed with 361 µl PCI (50 % buffer-saturated phenol, 48 % chloroform, 2 % isoamyl alcohol; Invitrogen), then gently inverted for 10 min until the mixture was homogenous. The suspension was transferred to a 1.5 ml phase-lock gel tube (Five Prime) and centrifuged for 5 min at 13 000 r.p.m. Afterwards, the upper aqueous phase was transferred to a low DNA-binding microcentrifuge tube. To precipitate the DNA, 0.1 volumes of 10 mM sodium acetate was added, followed by 0.54 volumes of 2-propanol. The suspension was allowed to sit at room temperature for at least 30 min then centrifuged for 30 min in a table-top centrifuge at 13 000 r.p.m. and 4 °C. After removal of the supernatant, 1 ml ice-cold 70 % ethanol was added and the suspension was centrifuged for 10 min at 13 000 r.p.m. and 4 °C. After removal of the ethanol, the DNA pellet was air-dried for 5 min to allow evaporation of excess ethanol. The DNA was resuspended in TE (Tris, EDTA) buffer and stored at 4 °C. More TE buffer was added as necessary until the DNA was fully solubilized. Agarose gel electrophoresis was carried out to assess the quality of the DNA before quantifying by Qubit.

### Tn-seq circle

The Tn-seq circle method [21] was used to enrich for the transposon–genome junction from the genomic DNA of transposon mutant libraries as described previously [21] with the modifications outlined here. Oligonucleotides used in this work are listed in Table S2. A sample of wild-type *B. cenocepacia* K56-2 was prepared simultaneously as a negative control. One hundred and thirty microlitres of approximately 38 ng µl^−1^ genomic DNA was fragmented to an average size of 300 bp via ultrasonication using a Covaris S220 (duty factor of 10 %, a peak incident power of 140, 200 cycles per burst, and a treatment time of four cycles at 20 s each). A-tailing was carried out on the purified end-repaired DNA in 50 µl reaction mixtures composed of end-repaired DNA, nuclease-free H_2_O, 5 µl Qiagen 10× buffer, 10 µl (1 mM) dATP, and 0.2 µl Taq (Qiagen) and incubated for 20 min at 72 °C in the thermal cycler. Size-selected DNA with ligated adaptors was digested with PacI (NEB) in 76 µl reaction mixtures containing 56 µl DNA, nuclease-free H_2_O, 7.6 µl Cutsmart buffer, and 1.9 µl PacI for 16–18 h at 37 °C. Size selection for 200–400-bp fragments was repeated, followed by DNA quantification. The cycles for the circularization reaction using Ampligase (Epicenter) were as follows: 30 s at 95 °C, 25 cycles of 30 s at 95 °C and 3 min at 67 °C, 3 min at 72 °C, followed by 2 min at 95 °C. The exonuclease treatment was carried out for 16 h at 37 °C. The enrichment of the transposon–genome junctions was confirmed by comparing the amplification of the HDTM library with wild-type *B. cenocepacia* K56-2 via real-time PCR using a Bio-Rad CFX Connect thermocycler. The optimal number of PCR cycles was determined using 10 µl reaction mixtures composed of template DNA, nuclease-free H_2_O, 0.02 µl (100 nM) of both primers 690 and 681, and 5 µl iTaq SYBRgreen Supermix (Bio-Rad) and cycled as follows: 3 min at 95 °C, 35 cycles of 30 s at 95 °C, 30 s at 63.5 °C, and 30 s at 72 °C. For amplicons created using KAPA polymerase (Kapa Biosystems), 10 µl reaction mixtures composed of template DNA, nuclease-free H_2_O, 0.2 µM of each primer 690 and 681, 0.1 µl of SYBRgreen (Life Technologies) 100× in DMSO, and 5 µl KAPA HiFi polymerase ready mix (Kapa Biosystems) were cycled as follows: 3 min at 95 °C, 35 cycles of 20 s at 95 °C, 15 s at 72 °C, and 30 s at 72 °C. The PCR was repeated using new reaction mixtures similarly prepared as before for the determined optimal number of cycles. The removal of primers containing the MiSeq adapter sequences from the amplicons was verified by running the sample on a Bioanalyzer (Agilent Technologies,). The purified samples were sequenced using the standard Illumina MiSeq standard kit v2 with 150-bp single reads at the Children's Hospital Research Institute of Manitoba (Winnipeg, Canada) following the manufacturer's instructions.

### Data analysis to determine gene essentiality

Gene essentiality was determined using modified versions of the custom scripts developed by Turner *et al*. [16] (see supporting data: https://github.com/khturner/Tn-seq/tree/dockerize). From the *B. cenocepacia* K56-2 gene feature format (gff) file, 10 % from the 3′ end and 10 % from the 5′ end was trimmed off of each gene so that insertions that are not likely to disrupt function are not included in the analysis. TnSeq2.sh (see supporting data: https://github.com/khturner/Tn-seq/tree/master/TnSeq2.sh) was used to identify reads containing the transposon sequence, remove the transposon tag and then map the remaining genomic sequences of the reads to the 17 contigs of the *B. cenocepacia* K56-2 draft genome (GenBank accession LAUA00000000) using Bowtie2 [52]. To avoid considering insertion sites that are over represented due to amplification bias, we discarded reads from the 100 most abundant insertion sites. To account for differential mutant abundance due to both genomic positional bias and PCR amplification bias we normalized the read data across the position of each contig, then based on G+C content of the region of insertion using the ‘lm’ function in R. We totalled the number of reads per truncated gene to calculate the normalized number of reads per gene as well as the number of insertion sites per gene. A Monte Carlo simulation then was run where insertion sites were moved randomly across the genome to generate an ‘expected’ pseudo data set. This simulation was repeated 2000 times. The Monte Carlo simulations provided an estimate of the variance that would be expected if there were no essential genes. Since it is possible to have a gene with no insertions when comparing the real and pseudo data sets, to avoid dividing by zero, one was added to the read count for each gene.

The R package EdgeR [53] was used to compare the variance of the pseudo data set with the mean read count per gene of the real data. To determine the differences of mutant abundance between the real and pseudo data sets, EdgeR uses a negative binomial test. Since multiple comparisons increase the rate of identifying false positives [54], to control the false discovery rate (FDR), the Benjamini–Hochberg method was used, which computes an upper bound for the expected FDR and adjusts the *P* value accordingly to correct for multiple testing [55]. The R MCLUST package [56] was used to perform bimodal clustering of genes to either a ‘reduced’ or ‘unchanged’ mode, by fitting a parameterized bimodal Gaussian mixture model to the log2-transformed fold change mutant abundance. A gene was classified as essential if it was significantly depleted in the real data compared to the pseudo data (adjusted *P*<0.05, negative binomial test), and clustered in the ‘reduced’ mode (*P*<0.05, maximum-likelihood estimation).

### Bioinformatics

The protein sequences from the 508 predicted essential genes of *B. cenocepacia* K56-2 were searched against all the bacterial essential genes in the database of essential genes (DEG 10, version 14.7, updated 24 October 2016) [57] using blastp with the default parameters provided in DEG and an E-value cut-off of 10^−10^. The essential homologues with the lowest E-value are listed in the Appendix. *B. cenocepacia* K56-2 homologues in *B. cenocepacia* J2315, [23] *B. thailandensis* E264 [48], and *B. pseudomallei* K96243 [22] were identified as the best hit from performing blastp using Geneious software with an E-value cut-off of 10^−10^, minimum 30 % sequence identity and 45 % coverage. Operon predictions and clusters of orthologous categories (COG) [58] were assigned to genes of *B. cenocepacia* K56-2 from the corresponding homologous gene in *B. cenocepacia* J2315 listed in the Database of prOkaryotic OpeRons (DOOR) [59].

## Results

### Production and sequencing of the HDTM libraries

With the goal of identifying the essential genome of *B. cenocepacia* K56-2, we used HDTM followed by Illumina sequencing of the transposon insertion sites. We generated a HDTM library of one million mutants by introducing the suicide plasmid pRBrhaBoutgfp [60] into *B. cenocepacia* K56-2 by triparental mating. To avoid insertions in non-essential genes from being lethal due to polar effects on downstream essential genes, the transposon contains a rhamnose-inducible promoter. Selection of transposon mutants in the presence of rhamnose allows expression of genes in a transcriptional unit downstream of the transposon insertion. To enrich for the transposon–genome junctions and identify the location of the insertion sites, we used the Tn-seq circle method [21] (Fig. S1).

As species of the genus *Burkholderia* have large multireplicon genomes with approximately 67 % G+C content [29], we first considered using a Hi-fidelity KAPA polymerase (KAPA bioscience) to amplify the transposon insertions by PCR. The KAPA DNA polymerase has been successfully used to increase the proportion of reads from transposon junctions in GC-rich regions in *B. thailandensis* [61] and has minimal amplification bias [62]. However, PCR-amplification of the transposon junctions may not be favoured by the KAPA DNA polymerase as the G+C content of the transposon sequence inserted into the genome is much lower (Fig. S2). To test the ability of the KAPA DNA polymerase to PCR-amplify the transposon junctions, the sequences of the HDTM library produced after PCR amplification with the KAPA DNA polymerase were compared with those obtained with the iTaq DNA polymerases (Bio-Rad). Sequencing the HDTM library after PCR-amplification with the KAPA polymerase resulted in 89 983 unique insertion sites with an average of 1 insert every 87 bases and a read G+C content of 61.0 % (Table 1). However, PCR-amplification with the iTaq DNA polymerase revealed 293 568 unique insertion sites, with an average of 1 insert every 27 bases, a read G+C content of 59.7 % (Table 1) and a lower proportion of insertion sites in GC-rich regions (Fig. S3). The total reads from PCR-amplification of the HDTM library with the iTaq DNA polymerase were more evenly distributed over the insertion sites, whereas the use of the KAPA DNA polymerase resulted in many insertions with a low read count and a large proportion of reads mapping to a small number of insertion sites (Fig. S4). For these reasons, the identification of essential genes was performed with the data produced using the iTaq DNA polymerase.

**Table 1.** Summary of results from sequencing the HDTM library

HDTM library preparation	Total reads	No. of reads containing the transposon sequence and mapping to the genome	G+C content of reads	No. of unique insertion sites	Frequency of Tn insertion
KAPA	20 459 975	4 370 457 (21 %)	61.0 %	89 983	1/87 bp
iTaq	15 132 067	6 936 891 (46 %)	59.7 %	293 568	1/27 bp

### Essential gene identification

**Fig. 1.**
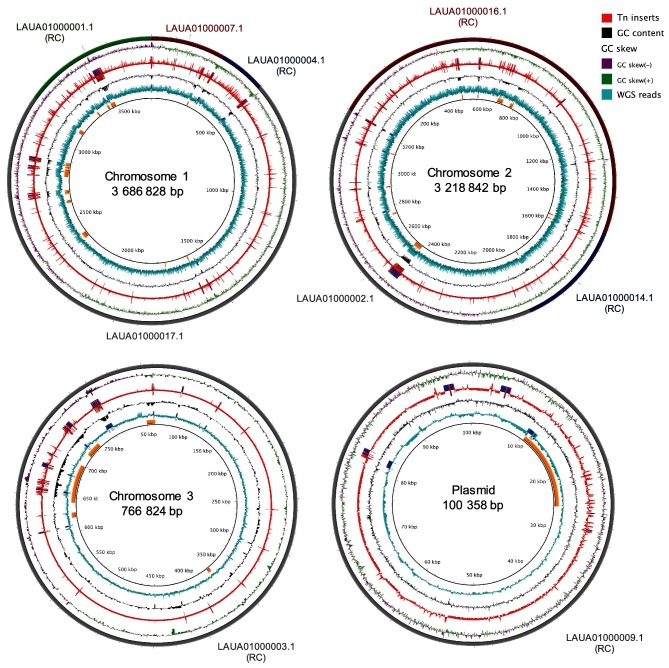
Distribution of reads in the HDTM library mapped to the *B. cenocepacia* K56-2 genome. From the outermost ring inwards. Ring 1: Contigs from the *B. cenocepacia* K56-2 assembly aligned to *B. cenocepacia* J2315 replicons using progressiveMauve [107]; RC, Reverse complemented. Ring 2: GC skew, positive (green), negative (purple). Ring 3: Alignment of reads from Tn-seq (Tn inserts, red). Ring 4: G+C content (black). Ring 5: Alignment of reads from the whole genome sequencing of *B. cenocepacia* K56-2 prepared with the Nextera XT kit (whole genome sequencing reads, turquoise). Rings 3 and 5: Genome regions with read coverage more than one standard deviation from the mean read coverage are indicated by blue regions. The innermost ring is the bp maker, with the genomic islands indicated by yellow arcs. Replicons are not to scale. Figure created using blast Ring Image Generator (BRIG 0.95) [108].

The first step in Tn-seq data analysis is to map the reads against the genome of the micro-organism under investigation (Fig. S5). As a complete genome of *B. cenocepacia* K56-2 is not available, we used the contigs from the draft genome of *B. cenocepacia* K56-2 [41] to map the Tn-seq reads and removed the 100 highest-read sites. After removing these reads, it was evident that there were positional effects on the insertion density and read counts (Fig. S6). These effects were not due to transposon insertion or Tn-seq method biases as the distribution of reads from sequencing the whole genome of *B. cenocepacia* K56-2 on the Illumina MiSeq platform exhibited the same trend. Notably, 17 of the 20 insertion sites with high read counts were in regions predicted to be genomic islands by Islandviewer3 [63] (Fig. 1). Overall, the reads were not evenly scaled across the contigs and the insertion density was strongly correlated with the G+C content (Fig. 1). To account for these biases, we created a model of read depth as a function of the position along each contig and G+C content. We then corrected the read count based on the model prediction to normalize the reads prior to the essentiality analysis, which minimized the effects of position and G+C content on read density (Fig. S7).

After normalizing the reads by contig and G+C content, we generated pseudocounts in 2000 simulations of randomly rearranging the read counts for each insertion site around the genome (Fig. S3). We then compared the mean read count from the actual data to the variance of the pseudocounts using the R package, EdgeR [53]. Essential genes were identified based on whether the mean read counts for a gene were statistically different than expected if the gene were not essential. Using this analysis, we identified 508 essential genes in *B. cenocepacia* K56-2 (Table S3). Included in our predicted essential gene set are genes in which disruptive mutations in *B. cenocepacia* causes a lethal phenotype: *dxs* (BCAM0911), *hemE* (BCAL0040), *infB* (BCAL1507), *gyrB* (BCAL0421), *ubiB* (BCAL0876), *valS* (BCAL1448), BCAL3369, and *murJ* (BCAL2764) [64–67]. In addition, we confirmed the essentiality of genes previously characterized by our laboratory. These genes encode EtfAB, an essential electron transfer flavoprotein [68] and EsaR, an essential response regulator involved in cell envelope integrity [69].

**Fig. 2.**
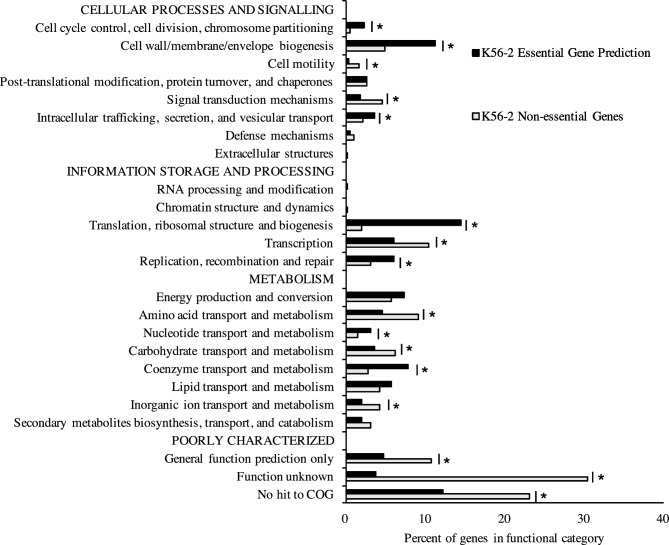
Functional categories of the predicted essential and non-essential genes of *B. cenocepacia* K56-2 Functional categories are based on the clusters of orthologous groups (COG) [58], which were assigned to genes of *B. cenocepacia* K56-2 from the corresponding homologous gene in *B. cenocepacia* J2315. Functional categories significantly enriched or under-represented in the essential gene set are indicated with an asterisk (*P*<0.05, Fisher exact test).

**Fig. 3.**
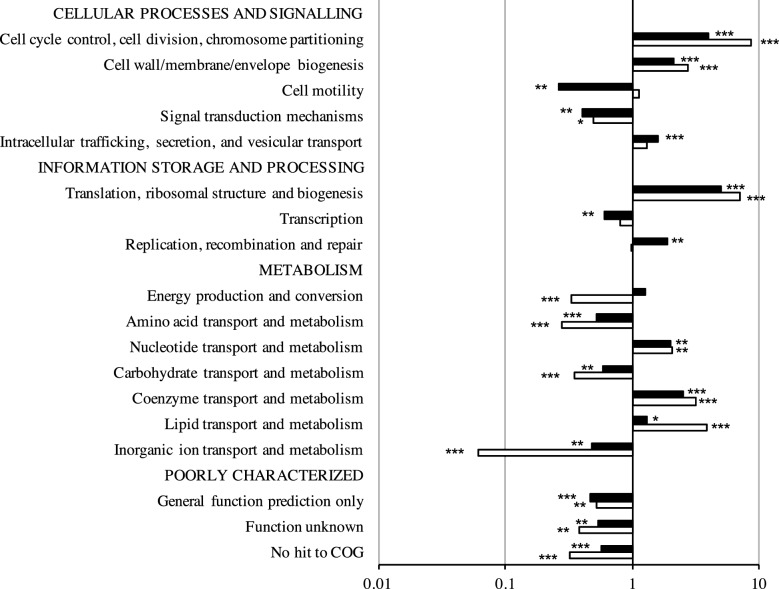
Comparison of the functional categories enriched or under-represented in the essential gene sets of *B. cenocepacia* K56-2 and *E. coli*. Enrichment and depletion of essential genes in COG categories compared to the representation of each genome were determined by Fisher’s exact test: *, *P*<0.1; **, *P*<0.05; ***, *P*<0.001. Black bars, *B. cenocepacia* K56-2; white bars, *E. coli* K-12 [10].

To identify which functional categories are represented in the essential genome of *B. cenocepacia* K56-2, we classified the essential and non-essential genes according to the cluster of orthologous group (COG) categories [58] previously identified for the K56-2 homologues in J2315 and listed in the Database of prOkaryotic OpeRons (DOOR) [59]. We assessed the enrichment of functions encoded by the predicted essential gene set for *B. cenocepacia* K56-2 with respect to non-essential genes in the genome. The essential gene set of *B. cenocepacia* K56-2 was enriched for genes involved in cell division, cell-wall functions, protein synthesis, replication, and co-enzyme metabolism (Fig. 2). Genes related to translation, ribosomal structure and biogenesis were the most highly enriched category in *B. cenocepacia* K56-2 (Fig. 2). The enrichment pattern observed was similar to that of *E. coli* K-12 [10] with the exceptions of three categories: (i) energy production and conversion, (ii) replication, recombination and repair, and (iii) cell motility (Fig. 3), possibly representing species-specific differences.

### Comparison of the essential genomes of *B. cenocepacia* K56-2 and J2315

**Fig. 4.**
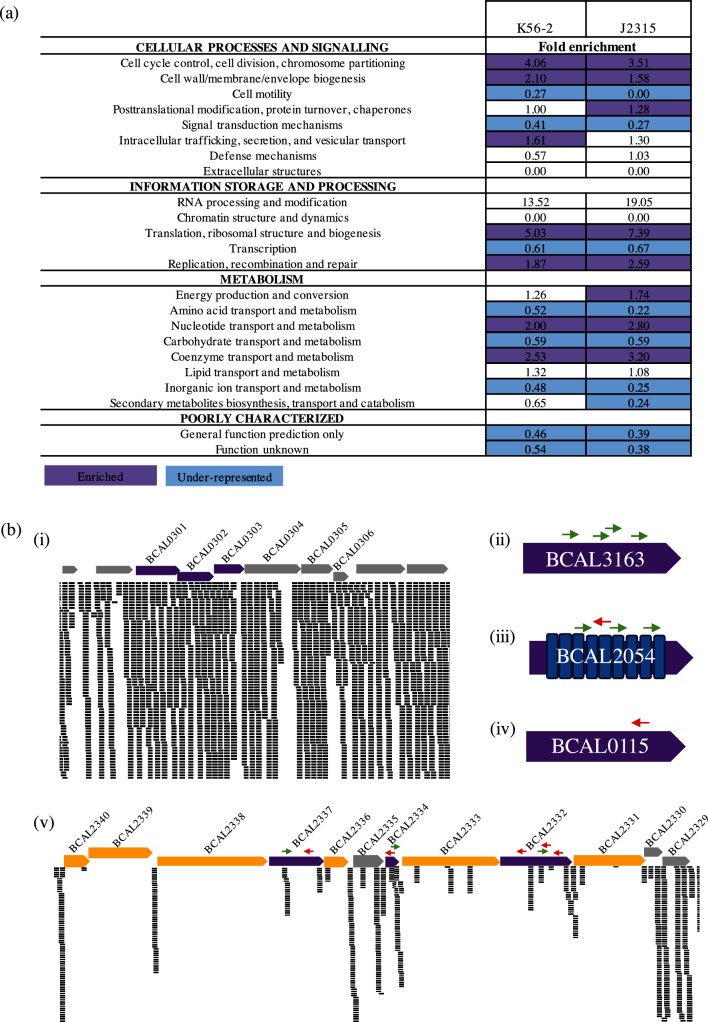
Comparison of the essential gene sets of *B. cenocepacia* K56-2 and *B. cenocepacia* J2315. (a) Fold enrichment of COG functional categories for the *B. cenocepacia* K56-2 and J2315 essential genes. Functional categories significantly enriched (purple) or under-represented (blue) in the essential gene are indicated (*P*<0.05, Fisher’s exact test). (b) Insertions and corresponding reads mapping to *B. cenocepacia* K56-2 genes identified as essential in strain J2315 but not in strain K56-2. Arrows represent the orientation of the P*rhaB* promoter (green, same direction as the ORF; red opposite direction to the ORF). (i) Operon BCAL0301–BCAL0306: BCAL0301, BCAL0302 and BCAL0303 (purple) were identified as essential in strain J2315, but had numerous insertions in strain K56-2. (ii) BCAL3163, encoding a putative nucleotidyltransferase, had four insertions in strain K56-2, all with P*rhaB* oriented in the same direction as the ORF. (iii) BCAL2054 encodes a protein involved in energy production and conversion that contains multiple phycobilisome (PBS) lyase HEAT-like repeat domains (IPR004155). (iv) Example of possible essential gene containing an insertion that disrupts within the last 35 % of the 3′ end of BCAL0115, encoding a 30S ribosomal protein S21. Only one transposon mutant was recovered containing the insertion disrupting the last 22 % of the 3′ end of the ORF. (v) Operon BCAL2340–BCAL2329 :  three genes were not identified as essential in either K56-2 or J2315 (grey), six were identified as essential in both K56-2 and J2315 (yellow), while three were identified as essential in J2315 only (purple). BCAL2332 has inserts disrupting the proton-conducting membrane transporter domain (PF00361). BCAL2334 has one insert with P*rhaB* in the same orientation as the ORF, and one insert disrupting the last 32 % of the 3′end with P*rhaB* in the opposite orientation of the gene. BCAL2337 had one insert disrupting 13 % of the 3′end with P*rhaB* in the opposite orientation of the gene and inserts disrupting 33 % from the 5′ end with P*rhaB* in the correct orientation for gene expression.

As a recent study reported the essential genome of *B. cenocepacia* J2315 [23], we predicted that the set identified by our study would overlap substantially with the one reported by Wong *et al*. given that strains K56-2 and J2315 belong to the same clonal complex [49]. However, while our predicted essential gene set consists of 508 genes, Wong *et al*. predicted 383 essential genes and 439 genes required for additional growth in liquid LB medium [23]. We first compared the enrichment of essential gene functions between the two strains of *B. cenocepacia*. The functions enriched in the essential gene set of K56-2 were similar to those enriched in the previously identified essential genes of J2315 (Fig. 4a). In contrast to *E. coli*, the essential gene sets of both K56-2 and J2315 involved in the replication, recombination and repair category are significantly over-represented, while genes involved in cell motility are under-represented (Fig. 4a). Although essential genes in the energy production and conversion category are more significantly enriched in strain J2315 (1.74-fold, *P*<0.05) than in strain K56-2 (1.26-fold, *P=*0.103) (Fig. 4a), these values are very different from those for the same category in *E. coli* (0.33-fold, *P=*0.103) (Fig. 3). Overall, essential genes are distributed across the same core biological functions in both strains of *B. cenocepacia*.

We next investigated the reasons for the discrepancy between the number of essential genes found between the two strains. From the *B. cenocepacia* K56-2 essential gene set, 294 genes have homologues to essential genes identified in *B. cenocepacia* J2315. Eighty-nine and 212 genes were uniquely essential to J2315 and K56-2, respectively. However, differences in the methodology of essential gene calling could cause this discrepancy. Our essentiality analysis used the read count per gene, which reflects the abundance of each transposon mutant in the library, whereas the analysis used to determine the essential genome of *B. cenocepacia* J2315 only considered the insertion sites per gene [15, 23]. We hypothesized that the 212 genes identified as essential in strain K56-2 but not in strain J2315 may correspond to genes with a strong effect on fitness. Therefore, these genes should be present in the set of genes required for growth after passage of the initial J2315 transposon mutant library through LB medium [23]. However, only 37 K56-2 essential genes out of 212 had homologues in J2315 that were identified as essential for survival in LB. In summary, from the 212 genes identified as essential in strain K56-2 but not in strain J2315, the essentiality of 175 genes appears to be specific to *B. cenocepacia* K56-2. In support of our findings, 107 out of these 175 genes have homologues that have been identified as essential in either *B. pseudomallei* or *B. thailandensis,* or have high similarity with essential genes found in the Database of Essential Genes (DEG, BLASTP, expect value cut-off 10^−10^) [57]. Ninety percent of the remaining 68 genes are annotated as hypothetical proteins (Table S3).

Of the 89 genes that are essential in strain J2315 but not in strain K56-2, eight do not have homologues in K56-2. For the remaining 81 J2315-specific essential genes that have homologues identified as non-essential in strain K56-2, we confirmed that 49 of the K56-2 genes contain high numbers of reads corresponding to multiple insertions throughout the gene [an example is shown in Fig. 4b(i)]. From the remaining 32 genes, 14 had significantly less reads (EdgeR adjusted *P*-value <0.05, negative binomial test) but could not be classified as essential with a high level of certainty (*P*>0.05, maximum-likelihood estimation). Next, we considered the possibility that some transposon insertions in non-essential genes in the J2315 transposon library may cause polar effects on downstream essential genes causing the transposon-disrupted gene to be identified as essential. In our study, 10 of these 32 genes had reads mapping to insertions within the internal 10–90 % of a gene and the rhamnose-inducible promoter (P*rhaB*) oriented to express the gene downstream of the insertion, three of which have a gene identified as essential in K56-2 directly downstream of the gene with P*rhaB* insertion [see example in Fig. 4b(ii)]. One gene, BCAL2054 had a similar insertion pattern, but also had an insertion where P*rhaB* is oriented in the opposite direction of the ORF [Fig. 4b(iii)]. BCAL2054 encodes a putative HEAT-like repeat protein involved in energy production and conversion. The insertions within BCAL2054 may not be disruptive, since it contains multiple phycobilisome (PBS) lyase HEAT-like repeat domains (IPR004155) [70]. For 21 genes identified as essential in strain J2315 but not in strain K56-2, our library contains mutants with insertions that only disrupt within the last 35 % of the 3′ end of the ORF [Fig. 4b(iv)], suggesting that only insertions into the 5′ end are disruptive. It is possible that this trend is also true for BCAL2332, BCAL2334 and BCAL2337 [Fig. 4b(vi), red arrows]. BCAL2334 and BCAL2337 had inserts within the last 32 % of the 3′end, while BCAL2337 had insertions disrupting the last 13 % of the 3′end and 33 % from the 5′. In addition, BCAL2334 and BCAL2337 had inserts with P*rhaB* in the same orientation as the ORF, allowing transcription of downstream coding regions [Fig. 4b(v), green arrows]. Taken together, from 89 genes identified as essential in *B. cenocepacia* J2315 but not in *B. cenocepacia* K56-2, 57 genes may correspond to actual strain-specific essential genes in *B. cenocepacia* J2315.

### Comparison of four essential genomes of *Burkholderia*

**Fig. 5.**
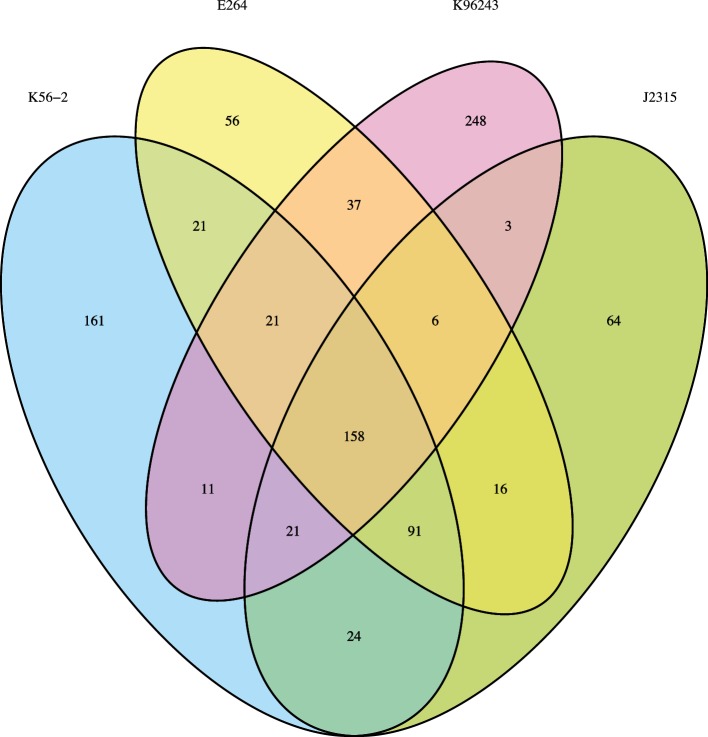
Comparison of the essential genes identified in *B. cenocepacia* K56-2, *B. cenocepacia* J2315 [23], *B. thailandensis* E264 [48] and *B. pseudomallei* K96243 [22] showing common and unique essential genes. Homologues were identified as the best hit from performing BLASTp using Geneious software with an E-value cut-off of 10^−10^, minimum 30 % sequence identity and 45 % coverage.

**Fig. 6.**
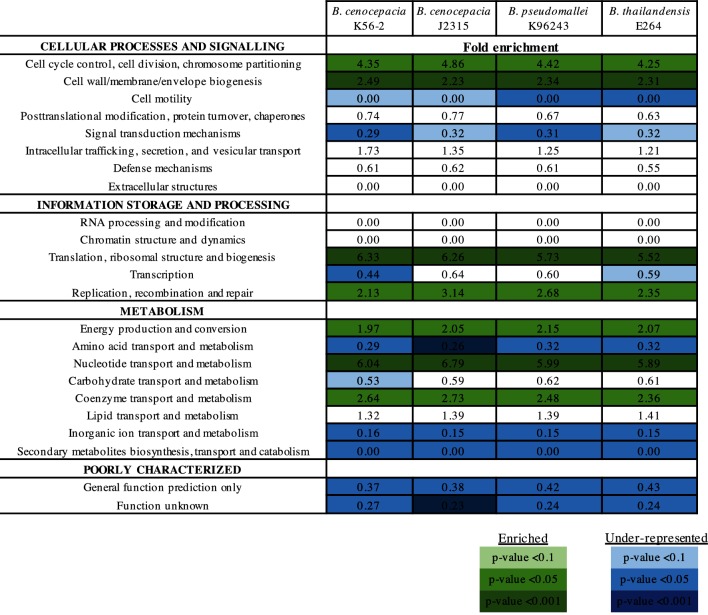
Functional categories enriched in the *Burkholderia* common essential genes. Fold enrichment of COG functional categories for the 158 essential genes common to *B. cenocepacia* K56-2, *B. cenocepacia* J2315, *B. pseudomallei* K96243 and *B. thailandensis* E264 compared to all the genes in each respective genome. Functional categories enriched (green) or under-represented (blue) in the essential gene sets are colour coded by *P*-value (Fisher’s exact test, legend).

**Fig. 7.**
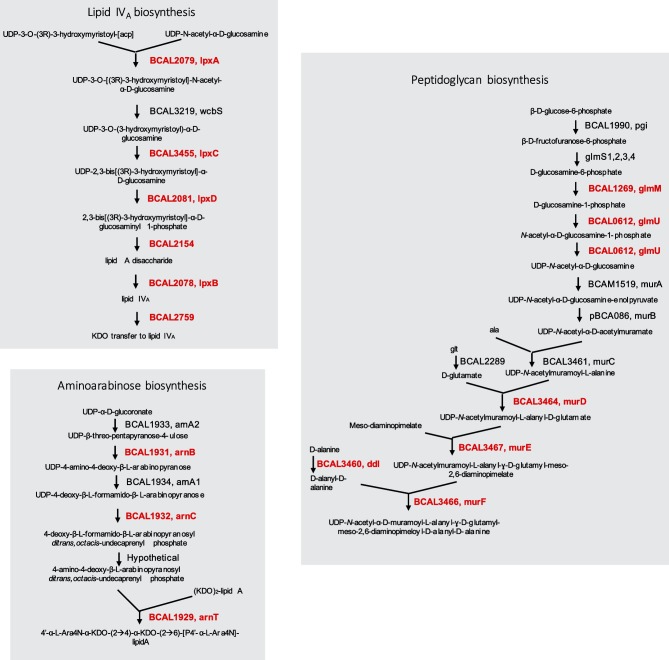
Pathways enriched in essential genes common to *B. cenocepacia* K56-2, *B. cenocepacia* J2315, *B. thailandensis* E264 and *B. pseudomallei* K96243. Pathways enriched in essential genes were determined using the pathway perturbation score (PPS) computed by BioCyc [71]. Red, essential genes,; black, non-essential genes.

The availability of essential genomes belonging to the two *Burkholderia* groups of human pathogens [22, 23, 48] allowed us to investigate conservation of essential genes with the goal of identifying common putative targets for antibacterial development. We found 158 genes that were commonly essential to the four *Burkholderia* strains (Fig. 5 and Table S4). The most significantly enriched essential functions of the 158 common essential gene set were translation, ribosomal structure and biogenesis, nucleotide transport and metabolism and cell wall/membrane/envelope biogenesis (*P*<0.001, Fisher’s exact test) (Fig. 6). In order to identify pathways enriched in essential genes common to the four *Burkholderia* strains, the 158 essential genes were mapped to *B. cenocepacia* J2315 pathways and a perturbation score (PPS) was computed using BioCyc [71]. The pathways of *B. cenocepacia* J2315 were scored to measure the extent of the enrichment of the 158 essential genes in a pathway by combining the essentiality of all reactions in the pathway. From essential genes identified as common to the four *Burkholderia* strains, we found that three pathways involved in the maintenance of the cell envelope, were highly enriched in essential genes: lipid IV_A_ biosynthesis, peptidoglycan biosynthesis and 4-amino-4-deoxy-arabinose (Ara4N) biosynthesis (Fig. 7).

Very recently, the essential genome of a non-ET12 clone of *B. cenocepacia* (H111) was published [72]. We compared the 158 *Burkholderia* common essential genes with the essential genome of *B. cenocepacia* H111 and found that all but 17 were conserved in this strain as well [72]. We then compared the unique insertion density scores (UIDs) of these 17 genes with the cut-off value (UID=0.01) Higgins *et al*. used to differentiate essential and non-essential genes. While all 17 genes had UID scores above the cut-off of 0.01, 16 had UID scores ranging from 0.0103 to 0.0290. While these genes may not be strictly essential in *B. cenocepacia* H111, fewer insertions in these genes are tolerated compared to the majority (94–89 %) of the genes in the genome, suggesting their importance for fitness. It is notable that the essential gene set of H111 also contains the genes involved in the pathways enriched in essential genes (Fig. 7).

While peptidoglycan and lipid A biosynthesis are essential in many Gram-negative bacteria [73, 74], Ara4N biosynthesis is not. Production of Ara4N is usually initiated in response to the presence of outer membrane stressors, such as the antimicrobial peptide polymyxin [75–78]. Ara4N replaces the phosphate groups of lipopolysaccharide (LPS), reducing the negative charge of the outer membrane and decreasing the affinity of cationic antimicrobial peptides [76]. Interestingly, polymyxin resistance is constitutive in many pathogenic species of the genus *Burkholderia* [79]. This finding led us to investigate whether there might be more essential functions in *Burkholderia* that are related to their particular biology. The amino acid sequences of the 158 common essential genes in *B. cenocepacia*, *B. thailandensis* and *B. pseudomallei* were then searched against all the strains in the DEG [57], excluding *Burkholderia*. This analysis identified three genes that are uniquely essential in the *Burkholderia* strains (BLASTP, expect value cut-off 10^−5^). These genes are annotated as encoding a polysaccharide deacetylase, a lysophospholipid transporter, LplT, and a general porin, Omp38 (OpcP). Homologues of these three *Burkholderia*-specific essential genes were also recently identified as essential in *B. cenocepacia* H111 by Higgins *et al*. [72]. Remarkably, the three genes encode cell-envelope-related proteins.

The putative polysaccharide deacetylase encoded by WQ49_RS30025 is an orthologue of that encoded by BCAL1935 in *B. cenocepacia* J2313 (reciprocal best hit, BLASTP), BTH_I2189 in *B. thailandensis* E264 and BPSL1468 in *B. pseudomallei* K96243 [70]. This protein is a member of the Ara4N biosynthesis gene cluster in *B. cenocepacia* J2315 and had been previously identified as essential in *B. cenocepacia* [67]. LplT functions to transfer membrane-disrupting lysophospholipids [80–82] across the inner membrane into the cell where they can be re-acylated by an acyltransferase/acyl-acyl carrier protein synthetase (Aas) [83]. *B. cenocepacia* K56-2 LplT (WQ49_RS29130) is an orthologue of the protein encoded by BCAL2111 in *B. cenocepacia* J2313, BTH_I2006 in *B. thailandensis* and BPSL2180 in *B pseudomallei* [70] (reciprocal best hit, BLASTP). Omp38 (OpcP) is a homologue of a major general porin, OmpF, in *E. coli* [84, 85] that is not essential for growth [10]. Omp38 (OpcP) is essential in *B. cenocepacia* K56-2 (WQ49_RS05300), *B. cenocepacia* J2315 (BCAM1931) [23] and *B. thailandensis* E264 (BTH_II1520) [70]. While the orthologue of Omp38 (OpcP) in *B. pseudomallei*, BPSS0879, (reciprocal best hit, BLASTP), was not identified as essential by Moule *et al*. [22], four putative porins were that have sequence similarity to Omp38 (OpcP): BPSL1655 (41 % identity, 95 % coverage), BPSL1674 (40 % identity, 94 % coverage), BPSL1728 (43 % identity, 95 % coverage), and BPSS0252 (34 % identity, 100 % coverage), and could possibly perform an analogous function to Omp (OpcP). Taken together these results suggest that protection of the outer membrane by surface charge modification and permeability modulation are uniquely essential functions in the three species of the genus *Burkholderia*.

## Discussion

In this work, one million mutants were collected and analysed to determine the essential genome of *B. cenocepacia* K56-2. The essential gene analysis was modified from the method of Turner *et al*., which identifies essential genes based on the relative abundance of reads mapping to each gene in the genome [16]. From our analysis, the most apparent bias was the high recovery reads and insertion sites corresponding to A/T-rich regions. Due to the low G+C content of the transposon sequence and subsequent reduced efficiency of PCR amplification, the use of KAPA polymerase did not improve recovery of insertion sites in GC-rich regions (Figs S3 and S4). Since the highest read counts were correlated with the G+C content and the read density mapping to each contig varied, we were able to minimize these biases by normalizing the reads to each contig (opposed to the proximity to the origin of replication) and to G+C content. (Fig. S7).

From sequencing a library of one million transposon mutants amplified using iTaq, we identified 293 568 unique insertion sites at an average of one insertion site for every 27 bp and 508 putative essential genes. Based on the number of essential genes in 14 bacteria with diverse genome sizes, we previously estimated the number of essential genes in *B. cenocepacia* to be 300–700 [60]. Putative essential genomes of three species of the genus *Burkholderia* have since been identified by HDTM [22, 23, 48, 72]. The identification of 508 essential genes is in agreement with the essential genes predicted for other species of the genus *Burkholderia*, including 406 in *B. thailandensis* E264 [48], 505 in *B. pseudomallei* K96243 [22] and recently, 383 essential genes were identified in a member of the epidemic ET12 lineage, *B. cenocepacia* J2315, which is closely related to *B. cenocepacia* K56-2 [23]. Within the *B. cenocepacia* K56-2 essential gene set, 291 and 211 have homologues to essential genes found in *B. thailandensis* E264 and *B. pseudomallei* K96243, respectively, while 296 have homologues to 294 essential genes identified in *B. cenocepacia* J2315. The high correlation with the functions represented by the *B. cenocepacia* J2315 essential gene set when grown in similar conditions as *B. cenocepacia* K56-2, as well as the identification of experimentally determined essential genes, give support to the validity of the essentiality analysis in *B. cenocepacia* K56-2.

We identified 212 and 89 genes that were uniquely essential in *B. cenocepacia* K56-2 and *B. cenocepacia* J2315, respectively. However, identification of these strain-specific genes could be due to differences in the methodologies used to generate the HDTM libraries and assign genes as essential in the two studies. *B. cenocepacia* strains K56-2 and J2315 were both mutagenized with a Tn5 transposon, however, in contrast to the triparental mating used in this work, the *B. cenocepacia* J2315 HDTM libraries were constructed by transforming electrocompetent *B. cenocepacia* J2315 with an *in vitro* transposition system [23]. While both methods involve a 2 h incubation before selecting for transposon mutants, competition among replicating cells is more likely to occur during the triparental mating than during electroporation, as the electroporated cells are fragile and are not expected to replicate. Depletion of transposon mutants with poor fitness would cause the disrupted genes in those mutants to be identified as essential, consistent with the identification of 37 genes as essential in *B. cenocepacia* K56-2 that were only required for survival in LB in *B. cenocepacia* J2315 (Table S3). The presence of essential genes downstream and in the same operon of a non-essential gene interrupted by a transposon insertion could also erroneously identify the interrupted gene as essential due to polar effects of the insertion. We considered that this could be responsible for the *B. cenocepacia* J2315 genes classified as essential by Wong *et al*. that we did not identify in *B. cenocepacia* K56-2, as their transposon did not contain an outward-facing promoter. However, although we did identify essential operons with different essential genes between the two strains of *B. cenocepacia* (J2315 and K56-2), these differences were not correlated with the gene order (Fig. 4b), indicating that polar effects of the transposons used in this work and by Wong *et al*. are not responsible for the differences with respect to genes within operons. Since non-lethal insertions in essential genes are possible when the insertion site is located near the 3′ or 5′ ends of a gene, we identified a gene as essential if there are not a significant number of reads mapped to the inner 80 % of the gene. However, insertions into the central portion of essential genes encoding multidomain proteins could be non-lethal if the insertion site is in the region outside of the essential domain [11, 86], as could be the case in BCAL2054 (Fig. 4b). Tolerance of insertions within the essential region of a gene could also occur due to a transient merodiploid state during replication, which could result in recovery of reads that map to an essential gene.

The genomic differences in the closely related isolates of *B. cenocepacia* could account for some of the discrepancies in the essential genes identified in *B. cenocepacia* K56-2 and *B. cenocepacia* J2315. For example, eight of the genes identified as essential in *B. cenocepacia* J2315 have no homologues in *B. cenocepacia* K56-2, and a 57-kb region encoding 57 genes is duplicated in *B. cenocepacia* J2315 (BCAL0969 to BCAL1026 and BCAL2901 to BCAL2846) but not *B. cenocepacia* K56-2 [40]. Fourteen genes in this region are essential in *B. cenocepacia* K56-2, most of which have homologues to essential genes identified in *B. thailandensis* and *B. pseudomallei* (Table S3). While 23.2 and 41.7 % of essential genes were identified as uniquely essential in *B. cenocepacia* J2315 and K56-2, a previous study showed that approximately 17 and 26 % of *Pseudomonas aeruginosa* PAO1 and PA14 essential genes, respectively, were uniquely essential for growth in CF sputum media [16]. The genomes of *Pseudomonas aeruginosa* PAO1 (6.26 Mb) and PA14 (6.54 Mb) are highly similar with strain-specific regions comprising 4.2 and 8.3 % of each genome, respectively [87]. Whether the essential genes that are distinct in *B. cenocepacia* K56-2 and J2315 are actually uniquely essential in each strain remains to be confirmed by further experimental evidence.

By comparing the essential gene set of *B. cenocepacia* K56-2 with *B. pseudomallei*, *B. thailandensis* and *B. cenocepacia* J2315, we identified 158 essential genes that are shared amongst the four *Burkholderia* strains. *B. thailandensis* and *B. pseudomallei* are genetically similar species that have different lifestyles and diverged from a common ancestor over 40 million years ago [88]. While *B. cenocepacia* belongs to the Bcc, *B. thailandensis* and *B. pseudomallei* belong to the *B. pseudomalle*i group. Both *B. cenocepacia and B. pseudomallei* cause infection in immunocompromised individuals. *B. pseudomallei* is classified as a category B biological threat agent [89] and is fatal in 10 to 40 % of melioidosis cases [90]. *B. thailandensis* rarely causes disease in humans [91], but can replicate in cultured mammalian cells and resists predation by amoeba [92, 93]. The finding of 158 shared genes between divergent strains highlights the importance of some essential processes in these pathogenic *Burkholderia* strains. One hundred and forty-one of these common essential genes were also recently identified as essential in the non-ET12 strain of *B. cenocepacia* H111 [72]. It is notable that 16 of the remaining 17 genes not identified as essential had fewer insertions per gene than approximately 90 % of genes in the genome of *B. cenocepacia* H111 [72], suggesting that these genes are important for growth in *B. cenocepacia* H111 as well. From the conserved essential gene set, we found that pathways involved in maintaining the integrity of the cell envelope are enriched in the common essential genes. Furthermore, we identified three genes that are uniquely essential in these four *Burkholderia* strains that are also involved in maintaining the integrity of the envelope.

BCAL1935 is transcribed with genes involved in l-Ara4N modification of lipid A, which has been previously identified as an essential process in *B. cenocepacia* [67]. BCAL1935 is included in a transcriptional unit that encodes the polysaccharide deacetylase and BCAL1936, which are predicted to synthesize UDP-Ara4N [67], however, the function of BCAL1935 is not known. While l-Ara4N-modified lipid A is required for resistance to antimicrobial peptides, it is not essential in most Gram-negative bacteria [76]. Recently it was discovered that in addition to l-Ara4N providing resistance to antimicrobial peptides, the presence of the l-Ara4N modification is required in order for LPS to be exported in *Burkholderia* strains [94], indicating how this modification is essential in *B. cenocepacia*.

The second gene identified as uniquely essential in the four *Burkholderia* strains encodes a lysophospholipid transporter (LplT) that transfers membrane-disrupting lysophospholipids across the inner membrane where they can be re-acylated by Aas [83]. LplT is not essential for growth in *E.coli* [10, 95] and the reason LplT is essential for growth in *Burkholderia* is unknown. However, conservation of the gene *lplT* would provide an advantage for surviving within a microbial community as well as within the host, which both employ phospholipases as a defensive mechanism.

We also identified a porin, Omp38 (OpcP), that is uniquely essential in three of the four *Burkholderia* strains. While OpcP is not essential in *B. pseudomallei*, four other essential porins, BPSL1655, BPSL1674, BPSL1728 and BPSS0252, were identified as essential [22]. Although none of the orthologues of the porins that are essential in *B. pseudomallei* were predicted to be essential in *B. cenocepacia* J2315 [23] or K56-2, it is possible that these porins provide a similar function to Omp38 (OpcP). The reason for the essentiality of the Omp38 (OpcP) porin is unknown, however trimeric porins like Omp38 (OpcP) contain binding sites that stabilize the LPS in the outer membrane in Gram-negative bacteria [96]. The outer membrane of species of the genus *Burkholderia* has been shown to be 89 % less permeable than *E. coli* [97] and the impermeability of the membrane in Gram-negative bacteria has been attributed to the functional characteristics of porins and LPS [76, 98].

Tn-seq of HDTM libraries provides a valuable resource for the identification of antibiotic resistance mechanisms [21, 99–101]. For example, clinical isolates of *B. pseudomallei* have been identified where loss of penicillin-binding protein 3 results in a high level of resistance to ceftazidime [102]. Future work passaging HDTM libraries in the presence of antibiotics has the potential uncover other resistance mechanisms. In addition, the presence of uniquely essential processes identified in this work suggest a role in survival specific the genus *Burkholderia*. However, further study of the essential genomes of other species of the genus *Burkholderia* is needed to confirm this hypothesis. Efforts are ongoing to find alternatives to conventional treatments for pathogenic species of the genus *Burkholderia* [103–106]. The envelope proteins identified in this work may represent ideal candidates for immunogenic targeting, as conserved essential proteins are less prone to variability.

## Data bibliography

1. Cardona ST. NCBI Sequence Read Archive (SRA) repository, accession number SRP112587 (2017).
2. Turner K. GitHub, https://github.com/khturner/Tn-seq/blob/master/TnSeq2.sh (2014).
3. Turner K. GitHub, https://github.com/khturner/Tn-seq/tree/dockerize (2017).

## Supplementary Data

1888 Supplementary File 1

## Supplementary Data

980 Supplementary File 2
