# Corrigendum: Comparative analysis of the *Burkholderia cenocepacia* K56-2 essential genome reveals cell envelope functions that are uniquely required for survival in species of the genus *Burkholderia*

**DOI:** 10.1099/mgen.0.000179

**Published:** 2018-05-31

**Authors:** April S. Gislason, Keith Turner, Mike Domaratzki, Silvia T. Cardona

**Affiliations:** ^1^​ Department of Microbiology, University of Manitoba, Winnipeg, MB, R3T 2N2, Canada; ^2^​ Monsanto Company, 700 Chesterfield Parkway W, Chesterfield, MO, 63017, USA; ^3^​ Department of Computer Science, University of Manitoba, Winnipeg, R3T 2N2, Canada; ^4^​ Department of Medical Microbiology & Infectious Diseases, University of Manitoba, Winnipeg, MB, R3E 0J9, Canada

There was an error in the text in the published article.

In the Discussion, the following text is incorrect: ‘BCAL1935 is included in a transcriptional unit that encodes the polysaccharide deacetylase and BCAL1936, which are predicted to synthesize UDP-Ara4N [67], however, the function of BCAL1935 is not known’.

It should read as ‘BCAL1935 is included in a transcriptional unit with BCAL1936. The product of BCAL1935 is a predicted deformylase (ArnD), which in *E. coli* removes the formyl group from undecaprenyl phosphate-4-formamido-l-arabinose [1]. The homolog of the BCAL1936 was characterized in B. cenocepacia K56-2 as a peroxiredoxin that confers resistance to oxidative stress [2].’

The following references should be used:

1. **Breazeale SD, Ribeiro AA, McClerren AL, Raetz CRH**. A formyltransferase required for polymyxin resistance in *Escherichia coli* and the modification of lipid A with 4-Amino-4-deoxy-l-arabinose. Identification and function of UDP-4-deoxy-4-formamido-l-arabinose. *J Biol Chem* 2005;280:14154–14167.

2. **Clarke DJ, Ortega XP, Mackay CL, Valvano MA, Govan JRW *et al.*** Subdivision of the bacterioferritin comigratory protein family of bacterial peroxiredoxins based on catalytic activity. *Biochemistry (Mosc)* 2010;49:1319–1330.

