# Supplementary material for: Comparative analysis of the Burkholderia cenocepacia K56-2 essential genome reveals cell envelope functions that are uniquely required for survival in species of the genus Burkholderia
Source: Microb Genom. 2017 Nov 21;3(11):e000140. doi: 10.1099/mgen.0.000140 (PMC5729917; doi:10.1099/mgen.0.000140)
Supplement: Supplementary File 1 [file mgen-3-140-s001.pdf]

Table S1. Bacterial strains and plasmids used in this study

	Features[a]	Source
**Strains**		
Burkholderia cenocepacia K56-2	ET12 lineage, CF isolate	Darling et al. (1998)
Escherichia coli SY327	*araD* Δ(*lac pro*) *argE* (Am) *recA56* Rif^r *nalA λ pir*	Miller and Mekalanos (1988)
E. coli MM290	F$^-$, φ 80 lacZΔM15 *endA1 recA1 hsdR17* ($r_H^- m_K^+$) *supE*44 *thi*-1 Δ*gyrA96* (*ΔlacZYA-arg*F) U169 *relA*1	Bloodworth et al. (2013)
**Plasmids**		
pRK2013	ori_{colE1}, RK2 derivative, Km^r *mob*$^+$ *tra*$^+$	Figurski and Helinski (1979)
pRBrhaBoutgfp	pSCrhaBoutgfp derivative (Cardona et al. 2006), ori_{R6K}, *rhaR rhaS PrhaB* e-*gfp*	Bloodworth et al. (2013)

*CF = cystic fibrosis, Km = kanamycin, Rif = rifampicin

Table S2. Oligonucleotides used in this study

Name	Sequence (5' - 3')*	Description
683	CCGTAGTGAGTTCTTCGTCCGAGCCACTCGGAGATGTGTATAAGAGACAGT	Top strand adaptor (has 3' T overhang) for Tn-seq circle
684	CTGTCTCTTATACACATCTCCGAGTGGCTCGGACGAAGAACTCACTACGG	Bottom strand adaptor for Tn-seq circle
682	CACAAGTGCGGCCGCACTAGTCTAGATTTAAATTACCGTAGTGAGTTCTTCGTCCGAGCCAC	Oligo probe for Tn-seq circle
690	AATGATACGGCGACCACCGAGATCTACACTAGATCGCTCGTCGGCAGCGTCAGATGTGTATAAGAGACAGNNNNAATCTAGACTAGTGCGGCC	Illumina Index N501 Tn-seq circle forward primer
681	CAAGCAGAAGACGGCATACGAGATTCGCCTTAGTCTCGTGGGCTCGGAGATGTGTATAAGAGACAG	Nextera Index N701 Tn-seq circle reverse primer

*N is any nucleotide.

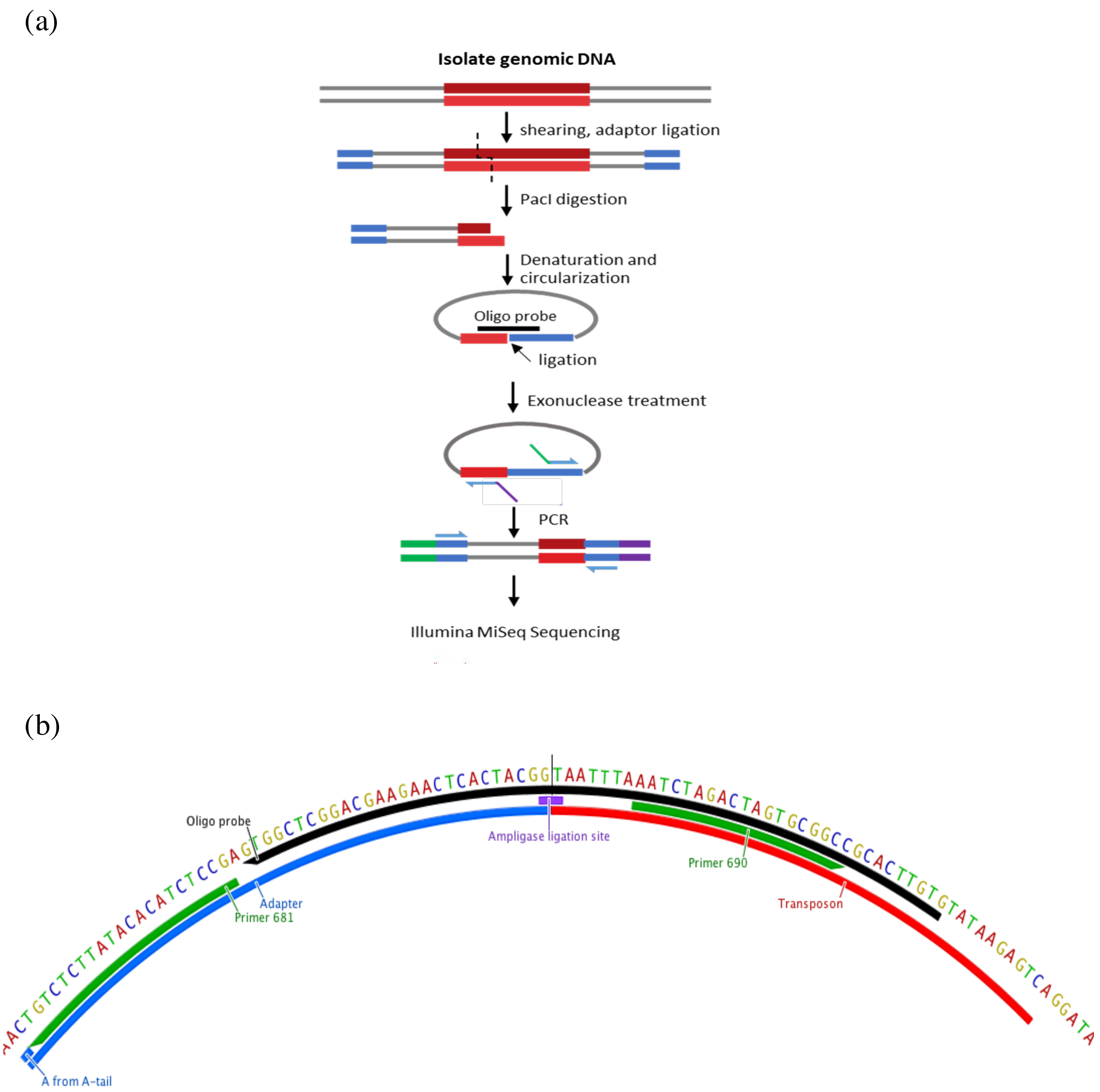

Fig. S1. Enrichment of transposon-genome junctions with Tn-seq circle.
A) Outline of the steps in Tn-seq circle. Adapters were ligated to genomic DNA that was sheared to an average size of 300 bp. Following PacI digestion, the DNA was denatured and annealed to an oligo probe. Only fragments containing the transposon and adapter sequences are circularized using the oligo probe. Exonuclease treatment degrades all linear DNA fragments, leaving behind fragments with the transposon-genome junction to be amplified by PCR prior to sequencing with Illumina MiSeq. Figure modified from Gallagher et al. (Gallagher, Shendure, & Manoil, 2011)
B) Example of circularization of a transposon mutant. Adapter, (annealed 683 and 684, Table S2), blue; transposon sequence, red; ligation site, purple; oligo probe binding site, (682, Table S2), red; primer binding sites, (690 and 681, Table S2), green.

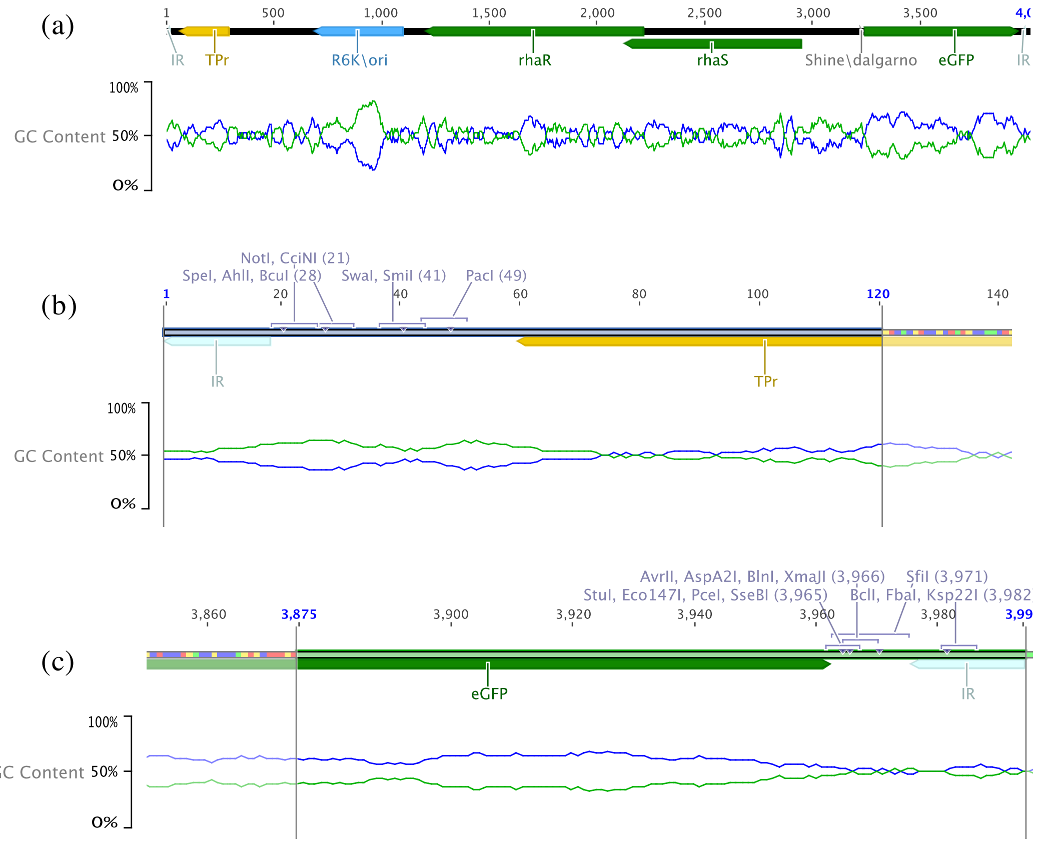

Fig. S2. GC content of the transposon insertion sequence is low.

A) The sequence of the transposon inserted into the genome, containing 51.3% GC content overall. B) The 5’ end of the transposon insertion sequence containing the pacI site. C) The 3’ end of the transposon insertion sequence containing the eGFP coding sequence located just downstream of P*rhaB*. GC(blue)/AT(green) content is indicated below each sequence. Sites of commercially available restriction enzymes that cut once within the insertion sequence are indicated.

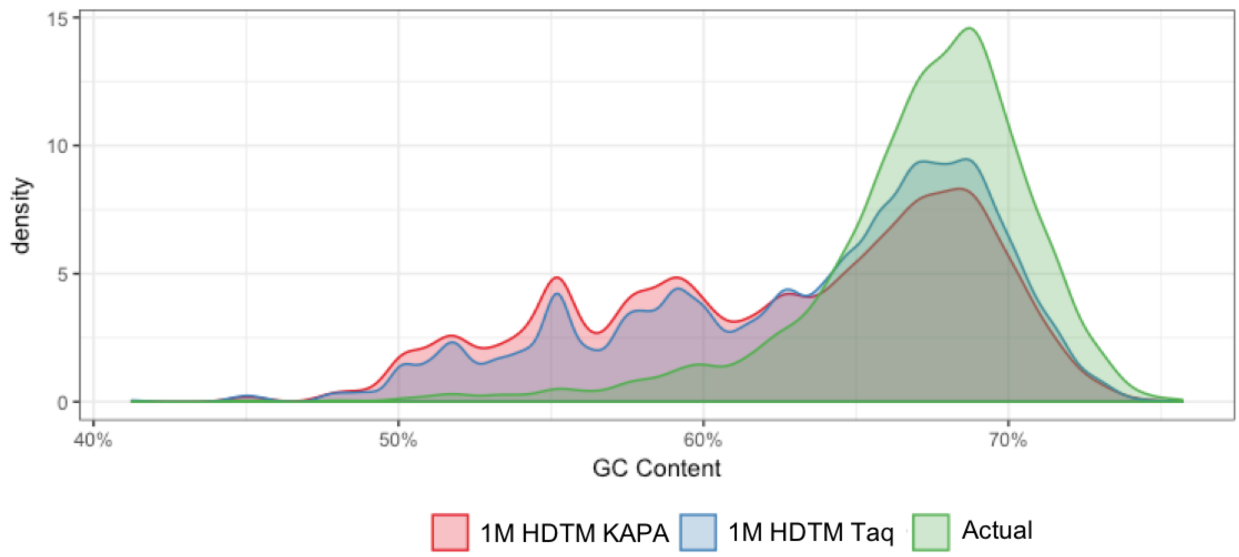

Fig. S3. Distribution of insertion sites in the genome by GC content.
A higher proportion of insertion sites in GC-rich regions were identified in the 1M HDTM Taq library, compared to the 1M HDTM KAPA library. Density of insertions sites (Y-axis) in the genome by GC content (number of insertion sites within a sliding window of 1 KB) scaled to a maximum value of 15; X-axis, GC content. Insertion site density of 1M HDTM KAPA library (pink) 1M HDTM Taq library (blue), actual GC content of the genome (green).

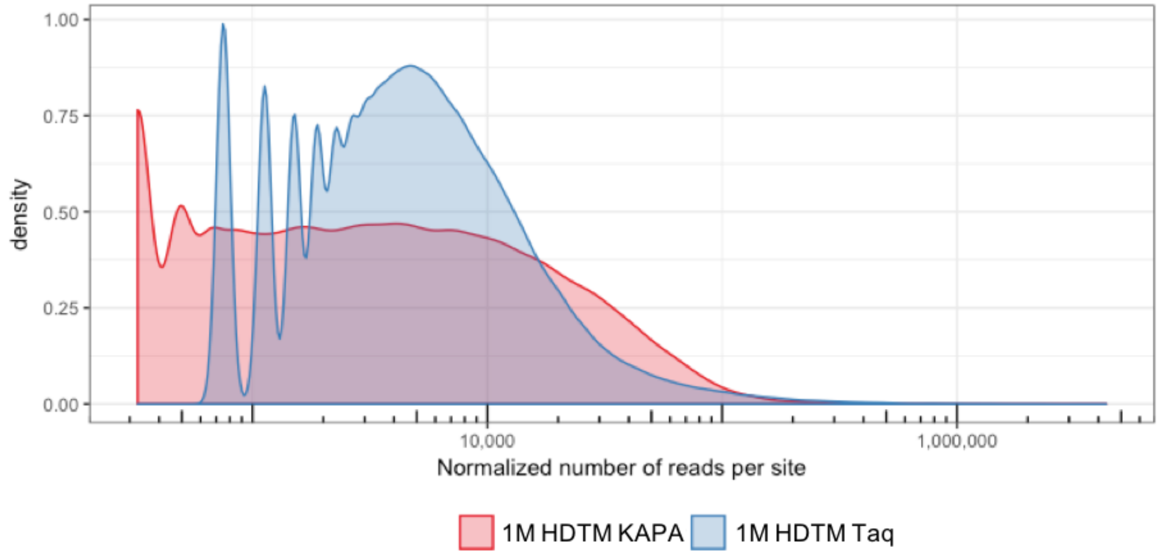

Fig. S4. Distribution of reads per insertion site
The frequency of reads per insertion site is shown for the HDTM libraries. The 1M HDTM KAPA library has a more skewed distribution of reads per site than the 1M HDTM Taq library. 1M HDTM KAPA, pink; 1M HDTM Taq, blue; Y-axis, density of insertions sites scaled to a maximum value of 1; X-axis, normalized number of reads per insertion site ($1x10^4$ x number of reads per insertion site divided by the mean number of total reads).

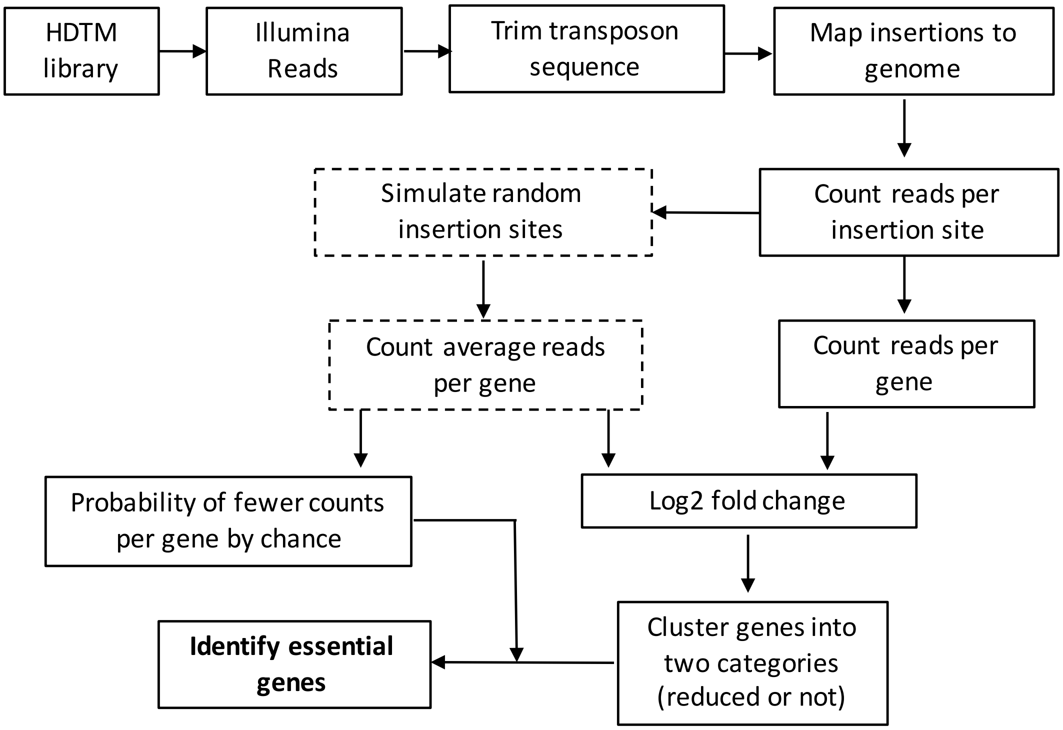

Fig. S5. Bioinformatics pipeline to identify essential genes
For the HDTM libraries, reads not containing a transposon sequence are discarded and the sequence is trimmed from the remaining reads. The trimmed reads are mapped against the reference genome using bowtie2 (Langmead, Trapnell, Pop, & Salzberg, 2009) and the position of each insertion and the number of reads mapping to it are collected. To identify essential genes using the HDTM library, the read counts are normalized to remove noise and position dependent differences. Then, simulations are run randomly moving the insertions throughout the genome as if there were no essential genes. For the experimental data and each simulated run, the number of insertion reads per annotated gene is collected, where insertions into the first 10% of the 5' end and last 10% of the 3' end of a gene are not considered disruptive. The log2 fold-change (experimental/mean simulated reads per gene) is calculated, as well as the probability of seeing fewer than the experimentally observed number of reads solely by chance.

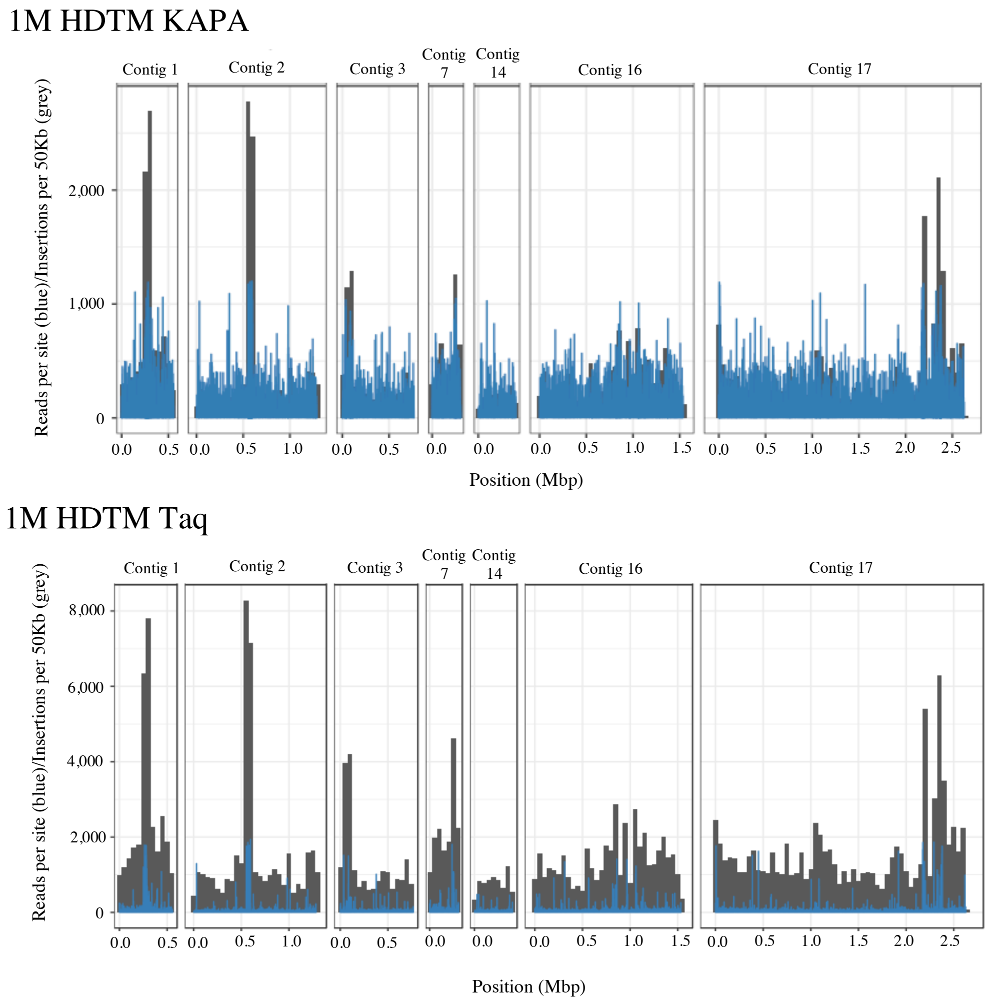

Fig. S6. Positional effects of insertion site density and reads per insertion.

The number of insertion sites (grey bars) mapping to the 7 largest contigs in a sliding window of 50Kb is indicated after the removal of the 100 insertion sites with the highest read counts (Y-axis). The read count per insertion site for each library is overlaid (y-axis, blue bars). X-axis, basepair position in each contig, 1M HDTM KAPA library (top), 1M HDTM Taq library (bottom).

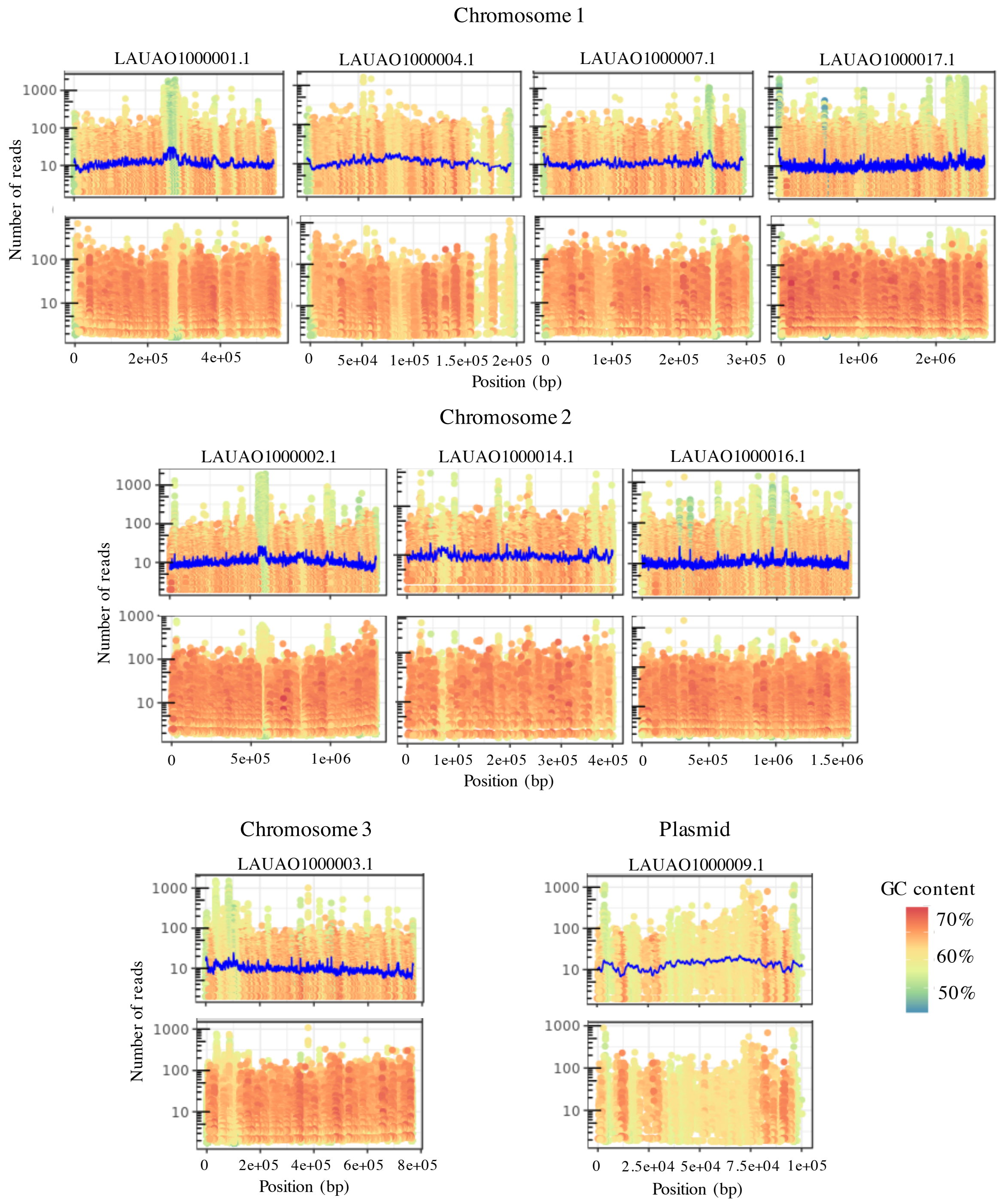

Fig. S7. Normalization of read counts from the 1M HDTM Taq library

Read counts mapping to each contig, arranged by replicon, and colour coded based on GC content (legend). Y-axis, number of reads, X-axis, base position in contig (bp). Top panels: Observed read counts for the contigs from each chromosome. The blue line represents the number of reads predicted from the model (read depth as a function of GC content and position along each contig). Bottom panels: Normalized read count after correction using the model prediction.
